# Editorial: The immune evasion and survival mechanisms in aquatic organism infections

**DOI:** 10.3389/fcimb.2025.1648166

**Published:** 2025-07-04

**Authors:** Einar Ringø, Su-Ming Zhou, Fei Yin

**Affiliations:** ^1^ Norwegian College of Fishery Science, Faculty of Bioscience, Fisheries and Economics, UiT The Arctic University of Norway, Tromsø, Norway; ^2^ School of Marine Sciences, Ningbo University, Ningbo, China

**Keywords:** bacteria, parasite, host, immunology, disease

Bacterial infections are the most common diseases in aquaculture and the common bacterial pathogens in aquaculture include *Aeromonas hydrophila*, *Aeromonas salmonicida*, *Vibrio*, *Edwardsiella*, *Streptococcus*, and *Flavobacterium* in fish and shellfish. These bacteria have been reported to cause huge economic losses in fish and shellfish farming worldwide (e.g., Toranzo et al., 2005; Maldonado-Miranda et al., 2022; Toffan et al., 2025).

Similarly, the presence of parasites has negatively impacted the reproductive capacity and survival of fish highlighting how external stressors can influence the physiological state of fish and their reproductive performance (e.g., Buchmann, 2022; Madsen and Stauffer, 2024).

The escalating utilization of antibiotics has resulted in a notable accumulation of antibiotic traces in the environment, and their detrimental effects on public health (e.g., Liang et al., 2013; Bilal et al., 2020; Sikder et al., 2024). The excessive and indiscriminate use of antibiotics leads to resistant microorganisms, complicating the treatment of infectious diseases, and the emergence and dissemination of antimicrobial-resistant genes have garnered significant attention from the global community (e.g., Salam et al., 2023; Sharma et al., 2024).

Based on these facts, it is of importance to improve secure strategies to control and prevent these diseases. Most aquatic pathogenic microbes exhibit robust immune evasion capabilities from the host immune system, and their evasion mechanisms aiding their survival in the target organs are the key to mediating the pathogenesis of these pathogenic microbes. When a pathogenic microbe (bacterium, virus or parasite) infects the body, a battle ensues between the host’s innate and adaptive immune systems and the pathogen’s assorted virulence mechanisms and factors occur. Understanding the underlying mechanisms that enable aquatic pathogenic microbes to evade from host immune system will offer novel and useful knowledge to help construct efficient therapeutic strategies for the prevention and treatment of correlated diseases (Hornef et al., 2002). Additionally, pathogen detection and monitoring in aquaculture by using molecular and technological are of importance (Rieder et al., 2025).

Aquatic animals mainly live in diverse environments, ponds, lakes, rivers, wetland, seawater and factory-style aquaculture facilities, which is more complex compared to those of terrestrial counterparts. Therefore, the causative pathogens species affecting aquatic animals, their adaptation mechanisms, and pathogenic mechanisms will significantly differ from those affecting mammal species. This Research Topic focuses on the evasion mechanisms and strategies employed by aquatic pathogens for immune evasion and host persistence.

In our invitation to the Research Topic “*The Immune Evasion and Survival Mechanisms in Aquatic Organism Infections*” we invited scientists to discuss the following sub-topics: a) virulence factors or molecules of the aquatic pathogens involved in invasion, persistence, or survival in aquatic animals, b) host-pathogen interactions or immune response to the aquatic bacterial and parasitic pathogens, c) strategies for overcoming the host innate immune response of the aquatic pathogens and d) mechanisms used by aquatic pathogens to evade the clearance mediated by the adaptive immune system. When the submission was closed five papers were published.

One study in the Research Topic “*The Immune Evasion and Survival Mechanisms in Aquatic Organism Infections”* by Teng et al. investigated proteome and transcriptome changes in the multiple signaling pathways involved in immunity in the northern snakehead (*Channa argus*) during *Nocardia seriolae* infection. Northern snakehead is a valuable aquaculture species in several Asian countries, but its productivity faces challenges, particularly diseases caused by *N*. *seriolae*, nocardiosis. To understand the modulation of the immune responses to *N. seriolae* infection in snakeheads, the authors evaluated the splenic proteome profiles. Totally 700 differentially expressed proteins (DEPs) were detected, 353 proteins exhibited upregulation, while 347 proteins revealed downregulation after *N*. *seriolae* infection. The DEPs were mapped in Kyoto Encyclopedia of Genes and Genomes database and displayed activation of several crucial pathways during infection. Among the pathways were ferroptosis, complement and coagulation cascades, chemokine signaling, tuberculosis, natural killer cell-mediated cytotoxicity, and Th17 cell differentiation. Construction of protein–protein interaction networks elucidated interplay between immune-related DEPs and these results revealed expression changes in multiple signaling pathways during the initial colonization phase of *N. seriolae*. The results of this study revealed novel insights into the infection mechanisms and host interaction dynamics associated with nocardiosis.

In a Chilean study, Mancilla et al. routine tested samples of *A. salmonicida* the etiological agent of furunculosis, a septicemic disease and demonstrated that the *vapA* locus is absent in a new strain involved in recent outbreaks in Chile with high mortality rates. *VapA* protein is the major membrane component, a critical virulence factor. Additionally, the authors reported that the *vapA*-absent strain differs from its counterparts in outer membrane protein and lipopolysaccharide profiles, suggesting profound changes at the membrane structure level and in antigenic properties. These features together with sequence analysis allowed the authors to suggest that a complex genomic rearrangement, probably an indel encompassing the entire *vapA* locus, gave rise to this membrane phenotype. Although pathogen evolution and emergence were not fully elucidated, the results suggest that the *vapA*-absent strain is responsible of recent furunculosis cases, and that the strain may be related to a less virulent disease as remarkable differences in virulence between *vapA*-absent and *vapA*+ isolates was noticed in intraperitoneal challenge as the *vapA*+ strain killed fish in a few days. Based on their results the authors put forward the hypothesis that the emergence of a new strain may be involved in recent outbreaks in Chile.

The Hong Kong oyster (*Crassostrea hongkongensis*) is of high economic and ecological value the coastal areas of the South China Sea. Additionally, the species is an ideal model for conducting scientific research on protection against pathogen infection and oxidative stress (Zhang et al., 2011; Xiang et al., 2014). ChPDIA3 encodes carboxypeptidase A3, a metalloproteinase primarily expressed in mast cells and plays a role in the degradation of proteins and inactivation of peptides, potentially involved in innate immunity and regulating the tissue microenvironment, and in the study of Hou et al. the highest expression of ChPDIA3 gene, using qPCR, was detected in gill tissue of Hong Kong oyster challenged to *V. harveyi*. Results showed that both miR-126-x and miR-21-y inhibited the 3’-UTR region of ChPDIA3, suggesting that both miR-126-x and miR-21-y have regulatory effects and inhibited ChPDIA3 expression.

The dimorphic fungi, *Candida albicans* (*C. albicans*) is a member of the normal human microbiota but causes a major portion of candidiasis cases in humans. As *C. albicans* forms biofilms (Mayer et al., 2013), a critical virulence factor that provides effective protection from commercial antifungals and contributes to public health. In the study of El-Gazzar et al., the authors isolated *Candida* spp. in 38 samples from Nile tilapia (*Oreochromis niloticus*), water and humans, which included 42% *C. albicans*. Totally 62.5% of the isolates were resistant to at least one antifungal agent, with the 62.5% resistance to nystatin, and 75% of the isolates were highly susceptible to amphotericin. All *C. albicans* isolates exhibited biofilm-forming capabilities, and 4 isolates showed strong biofilm formation. One virulence associated gene (RAS1, HWP1, ALS3, or SAP4) was identified among the *C. albicans* isolates. Furthermore, the authors investigated the antifungal and antibiofilm effects of probiotic *Lactobacillus salivarius* (*L. salivarius*), zinc nanoparticles (ZnNPs) and nanocomposites (ZnNCs) on *C. albicans* isolates and showed that they displayed antibiofilm and antifungal effects against C. albicans, with highest inhibitory activity by ZnNCs. Additionally, scanning electron microscopy images of *C. albicans* treated with ZnNCs revealed asymmetric, wrinkled surfaces, cell deformations, and reduced cell numbers.

The myxozoan parasites, *Myxobolus cerebralis*, and *Tetracapsuloides bryosalmonae* cause severe disease of salmonids. Whirling disease is caused by *M. cerebralis* (Hofer, 1903; Hoffman, 1990), while proliferative kidney disease is caused by *T. bryosalmonae* (Okamura et al., 2011). As little is known about the proteomic changes at the portals of entry in rainbow trout after infection with *M. cerebralis* and *T. bryosalmonae*, Saleh et al. wanted to provide information whether single and coinfection with *M. cerebralis* and *T. bryosalmonae* modulated proteomic changes in the caudal fins and gills of rainbow trout before and after co-infection, using a quantitative proteomic approach. The results showed that in the caudal fins, 16 proteins were differentially regulated post exposure to *M. cerebralis*, while 27 proteins were differentially modulated in the gills of the infected fish post exposure to *T. bryosalmonae*. In the caudal fin, after co-infection, four proteins involved in parasite recognition and the regulation of host immune responses were differentially modulated between the groups. In the gills, 11 proteins involved in parasite recognition and host immunity, including four myxozoan proteins predicted to be virulent factors, were differentially modulated.

The studies cited above showed interesting results for the scientific community and the global community, but to conclude, the studies in this Research Topic highlighted the importance of additional studies. Fish are considered lower vertebrates with an underdeveloped immune system, requiring activation or induction methods to enhance the immune resistance against pathogens. Antigen-presenting cells play a role in promoting adaptive immune responses and enhancing the host’s immunity. By isolating and culturing antigen-presenting cells and preparing recombinant anchor proteins from pathogens, researchers aim to explore the immune recognition role and patterns of fish APC surface receptors in recognizing pathogen recombinant anchor proteins, thereby revealing the antigen recognition mechanisms of APCs in fish adaptive immune responses. This will provide a theoretical foundation for further research on the adaptive immune response mechanisms in bony fish.

A topic that merits investigation is that climate change provides more suitable conditions for myxozoan parasites lifecycle, which may lead to decline of wild trout populations in North America and Europe.
